# High-throughput automated home-cage mesoscopic functional imaging of mouse cortex

**DOI:** 10.1038/ncomms11611

**Published:** 2016-06-13

**Authors:** Timothy H. Murphy, Jamie D. Boyd, Federico Bolaños, Matthieu P. Vanni, Gergely Silasi, Dirk Haupt, Jeff M. LeDue

**Affiliations:** 1 Department of Psychiatry, Kinsmen Laboratory of Neurological Research, University of British Columbia, Vancouver, British Columbia, Canada V6T 1Z3; 2 Djavad Mowafaghian Centre for Brain Health, University of British Columbia, Vancouver, British Columbia, Canada V6T 1Z3

## Abstract

Mouse head-fixed behaviour coupled with functional imaging has become a powerful technique in rodent systems neuroscience. However, training mice can be time consuming and is potentially stressful for animals. Here we report a fully automated, open source, self-initiated head-fixation system for mesoscopic functional imaging in mice. The system supports five mice at a time and requires minimal investigator intervention. Using genetically encoded calcium indicator transgenic mice, we longitudinally monitor cortical functional connectivity up to 24 h per day in >7,000 self-initiated and unsupervised imaging sessions up to 90 days. The procedure provides robust assessment of functional cortical maps on the basis of both spontaneous activity and brief sensory stimuli such as light flashes. The approach is scalable to a number of remotely controlled cages that can be assessed within the controlled conditions of dedicated animal facilities. We anticipate that home-cage brain imaging will permit flexible and chronic assessment of mesoscale cortical function.

## Abstract (web summary)

Functional imaging in awake head-fixed mice is a widely used technique to study neural responses. Here the authors report on an open source, fully automated unsupervised system for training mice to self initiate head fixation to enable stable mesoscopic functional imaging of cortical functional connectivity.

## Graphical abstract

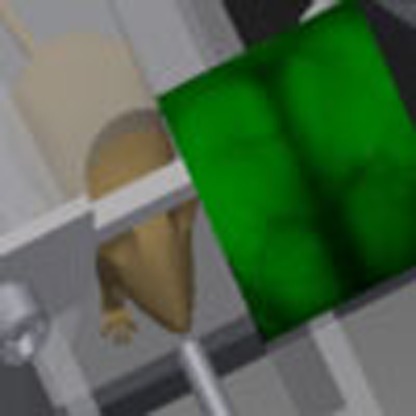

The cortex is organized into mesoscale maps^1^ that reflect sensory stimuli from the external world, or the planning and execution of cognitive and motor function. Wide-field imaging of cortical activity^2^,^3^,^4^,^5^,^6^,^7^,^8^,^9^ provides an opportunity to assess circuit action with near-real-time resolution. Recently, the development of genetically encoded calcium indicator (GECI) transgenic mice has provided a new selective and sensitive means for assessment of brain activity^10^. These animals, combined with relatively noninvasive transcranial imaging^11^,^12^, enable longitudinal assessment of cortical activity. Although imaging methodology and probes have advanced considerably^13^, awake animal *in vivo* imaging requires significant investigator involvement at both training and assessment stages. Recent developments in fibre optics and implantable microsensors offer the ability to make small field-of-view measurements, or point photostimulation with extensions to home cages^14^, but do not permit the assessment of multiple animals or wide fields of view. Head-fixed mice provide a means of obtaining high-resolution imaging data through a variety of different brain window preparations^11^,^12^,^15^,^16^. Immobilization may also constrain possible behaviours, which can simplify interpretation of results. Disadvantages of head-fixed assessment are potential for animal stress and considerable time investment by investigators to perform the process.

Previous work^17^ has defined an important training paradigm for self-head-fixation with minimal investigator involvement and some degree of control by animals in two-photon (cellular level) imaging of rat cortex. In the case of rat automated head-fixation, the animals were able to release themselves at will and preferred short-duration trials of fixation that were less than 10 s (refs 17, 18). We extend previous self-initiated rat brain imaging^17^,^18^ to study multiple transgenic mice and lines^10^ within home-cage housing (see Supplementary Video 1) using inexpensive imaging and control hardware (Picam and Raspberry Pi) that can be controlled remotely via a secure shell protocol.

Our focus has been on developing a platform for millimetre-scale mesoscale wide-field imaging^1^ of interactions between cortical areas. We extend previous self-head-fixed rat imaging using two-photon microscopy^17^ to a multi-mouse, home-cage-based system that resolves regional activity over mesoscopic spatial scales and can be maintained autonomously within an animal facility.

## Results

### A system for automated head-fixation within mouse home-cages

**Figure 1:**
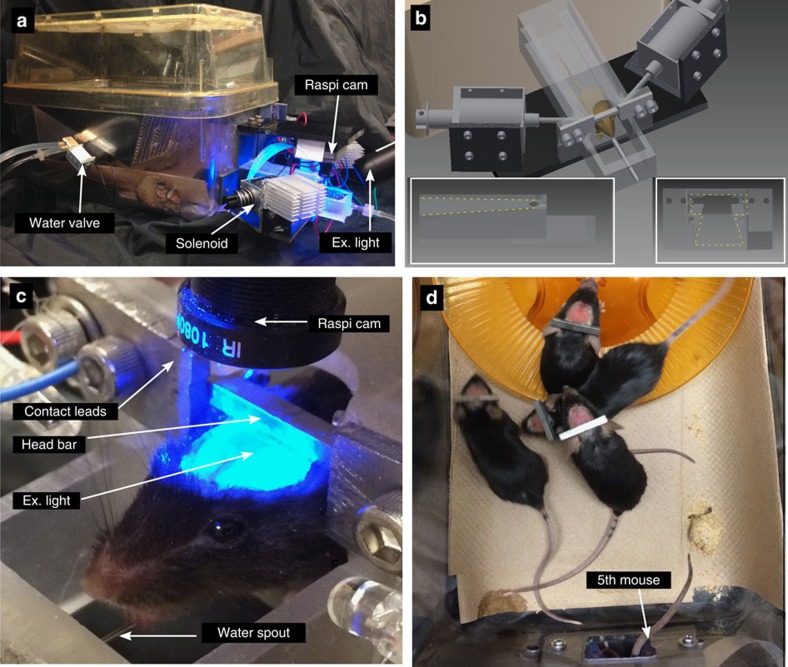
Self-initiated high-throughput home-cage mouse cortical imaging. (**a**) Mouse cage with auto-head-fixing device and camera for GCaMP6 imaging attached (note RFID reader is underneath the chamber so it is not visible) (**b**) CAD drawing of auto-head-fixing device showing (partially transparent) fixation tube and tracks in which mouse directs head fixation bar (insets left and right respectively show side and front view of the track). Solenoid-driven pistons for bar fixation and metal plates for contact detection are shown. (**c**) Close-up photo of auto-head-fixed mouse showing head fixation bar, camera, waterspout and fixation plates. (**d**) Photo of four mice near the exercise wheel (orange) within home cage with auto-head-fixing device while a fifth mouse is head fixed within the fixation tube at the bottom of the image (only tail is visible).

Using a standard mouse housing cage of 28 × 18 × 12 cm, we attached a three-dimensional (3D)-printed head-fixing chamber (Fig. 1a,b). The opening of the chamber is shaped such that it only permits adult mice with 22.2-mm-wide steel head bars to enter if the bar is aligned with the overhead tracks (outlined in yellow, Fig. 1b inset left and right). The track is tapered toward the end of the chamber so that as the animal progresses the bar becomes more tightly fitting within the track, reducing twisting. At the end of the track, the head bar makes electrical contact with two steel plates, which engage solenoid-driven pistons (see parts list, Table 1) to provide reproducible head-fixation of home-cage mice (see Supplementary Video 2). The 24 V push solenoids have a linear lift capacity of 1.2 kg. Given that the solenoids make contact with the bar at a 30-degree angle, we estimate the holding force to be 5.9 N or less. Once mice are head-fixed, we image brain activity through a bilateral transcranial window encompassing much of the cortex^3^,^12^ for 30–64 s epochs (Fig. 1c), and in some cases longer sessions. The imaging area is ∼9 × 9 mm and occurs through intact bone. During head-fixation, the animals were rewarded with access to water through a spout (blunted 22-gauge needle) placed ∼15–17 mm anterior and 12 mm below the location of the head-fixation bar. The fixation bar (22.2 × 2.7 × 3.2 mm) was placed ∼4 mm posterior from the centre of the bregma landmark and was made of chromoly steel (McMaster Carr, 4468T11). The fixation bar was a simple rectangle allowing them to be produced in a standard machine shop with minimal labour. Multiple mice are shown with fixation bars in Fig. 1d. To secure the bar to the skull, Metabond dental adhesive was used.

To control image acquisition and the head-fixing trials, we used the Raspberry Pi model 2 single-board computer and its camera module. Improvements in consumer camera technology, as well as significantly larger fluorescence responses for GECIs^10^, have made it possible to monitor brain activity using relatively inexpensive sensors such as the raspi cam (∼$27 USD). Given these larger GECI signals, artifacts associated with hemodynamics^12^, flavoprotein signals^6^, or movement, make a relatively smaller contribution. The scripts were written in Python to measure fixation bar contact, engage fixation, open water delivery valves and initiate image acquisition (See the block diagram of the hardware in Supplementary Fig. 1).

To acquire images of GCaMP6 cortical signals from Ai94 or Ai93 head-fixed mice, we used the picamera Python library and the (Wave Share Electronics RPi Camera (F)) Raspberry Pi camera (raspi cam; Fig. 1c).A plastic adjustable lens (*f*=3.6 mm; provided with the camera) was used after unscrewing the lens and placing a 10-mm-diameter green emission filter (ET525/36m, Chroma Technology) between the lens and the imaging sensor. The use of this camera and lens resulted in a bilateral 10.5–10.75 × 10.5–10.75 mm field of view and collection of video files in H264 or raw RGB24bit format.

By using implanted RFID tags (125 kHz glass Sparkfun) and a tag reader (ID-20LA RFID Sparkfun), we independently monitored the brain activity of up to five mice per cage 24 h per day. The tag reader was placed under the fixation tube and only registered entries if a mouse placed most of its body within the tube. Entry and exit tag times were used to associate image sequences with particular mice.

### Automated head-fixation and mesoscale imaging

**Figure 2:**
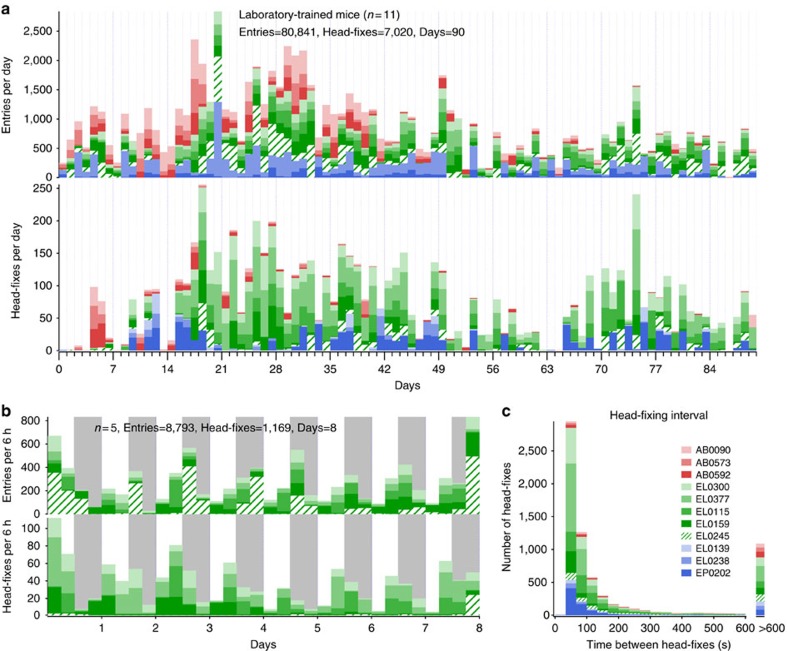
Laboratory-trained mice frequency of head-fixing and entrances. (**a**) Time course showing daily totals for entries and head-fixes for 11 mice represented in different colours. Two cages of three mice EP (shown in shades of blue) and AB (red) and one of five mice EL (green); all mice were head-fixed. (**b**) Graph showing frequency of head-fixation for five unsupervised mice assessed 24 h per day for 8 days in 6-h bins (grey shading indicates night). (**c**) Measure of repeat head-fixing frequency determined from the time interval between head-fixes. Data from 11 mice in cage groups named EP, EL and AB are shown. Most repeated head-fixes occur within minutes of a previous head fixation.

A total of 16 female mice were trained for automatic head fixation: two GFP-m (controls for nonspecific signals), 11 GCaMP6s (Emx1-cre;CaMK2-tTA;Ai 94) and 3 GCaMP6f (Emx1-cre;CaMK2-tTA;Ai 93) and followed daily up to 3 months. Preliminary experiments were performed on three male mice; two male mice were successfully head-fixed in a preliminary version of the cage. After observing the males, we chose to work with only females since males are potentially more aggressive and do not offer the ability to combine different litters to perform cross-genotype within cage assessments. Furthermore, head-fixation puts mice in a potentially vulnerable position with respect to injury from cage-mates. Over a 3-month period, a group of 11 mice (organized into cage group subsets named EP, EL and AB) collectively initiated 7,020 30-s sessions of head-fixed brain imaging (Fig. 2a). Eleven female mice were head-fixed on average 7.1±6.6 times per day, but entered the tube on average 81.7±23.9 times per day obtaining both ‘entrance rewards' that did not require fixation and head-fixed rewards (Fig. 2a and Table 2). A cage of five fully trained mice for automatic head-fixing was followed continuously for an 8-day period within the laboratory (subset cage EL). These mice were free to voluntarily initiate self-head fixation 24 h per day and did so for a total 1,169 unsupervised sessions of cortical imaging (Fig. 2b). Although these numbers seem considerable, they are still well less than auto-head-fixation rates of rats where 500 trials per rat can be performed in a day^18^. As evidence that the procedure was not aversive, we observed that mice would voluntarily head-fix repeatedly within 60 s (Fig. 2c and Supplementary Video 2). To reinforce head-fixed behaviour, we gave relatively more water per trial for head-fixation than just tube entry (up to 30 ×). In some cases, we placed a maximum on the number of entrance water rewards available without head-fixation (∼50–75% of minimum daily water intake of 1 ml). With the exception of an 8-day period when the mice were followed for 24/7, these mice (EP, EL and AB cages) were studied in daily 2–6 h sessions within their head-fixation cage that was placed on a laboratory bench. At the end of daily training within the laboratory, all the mice were weighed and given supplementary water on the basis of their weights and were returned to their animal facility where they were water restricted. Within these head-fixing cages, there was variability in rates between mice, 4 of 16 mice head-fixed greater than 10 times per day, whereas others head-fixed as few as two times and one mouse not at all (Table 2). It should be noted that these head-fixing stats include the initiation of training as well as perturbations that included lengthening of trials and round the clock randomly triggered visual stimuli. Although mice head-fix less times than rats^18^, the durations of mouse head-fixation was at least four to eight times longer than the rat work and have the potential to be extended to several minutes. Given that we use female mice, it would be possible to pool together mice (from multiple cages) that are more likely to head-fix creating a cage where all the animals would be expected to have high rates.

### Daily laboratory and 24 h per day unsupervised training

The animals were self-trained by first giving unlimited water from small water bottle placed within the training tube of a dummy home cage that lacked electronics or solenoid pistons (Supplementary Fig. 2). Over time, we moved the position of the water bottle so the mice had to venture further within the tube and weighed the bottle daily to confirm consumption (average consumption per mouse 2.7±1.4 ml per day; *n*=5 mice). After 7–10 days, the mice were drinking from the water bottle spout at the end of the fixation tube, but still had unlimited access to water (see ‘Methods' section for detailed training and Supplementary Fig. 2). The mice were then switched to a head-fixing cage that contained the RFID tag reader and a water spout made from blunt 22-gauge needle tubing. Water -deprived mice (for 24 h) were then given water automatically for entering the tube (indicated by their RFID tag), but initially placed the water spout so that the mouse could easily reach it without engaging the head-fixation circuitry (5–10 mm past the end of the tapered track). Over time, the water spout was moved to a final position that was 15–17 mm past the end of the track for all the mice. Eleven of 16 mice were trained in the laboratory on a bench-top in daily minimally supervised sessions of 2–6 h in the head-fixing cages over periods up to 12 weeks (see ‘Methods' section and Fig. 2a–c).

**Figure 3:**
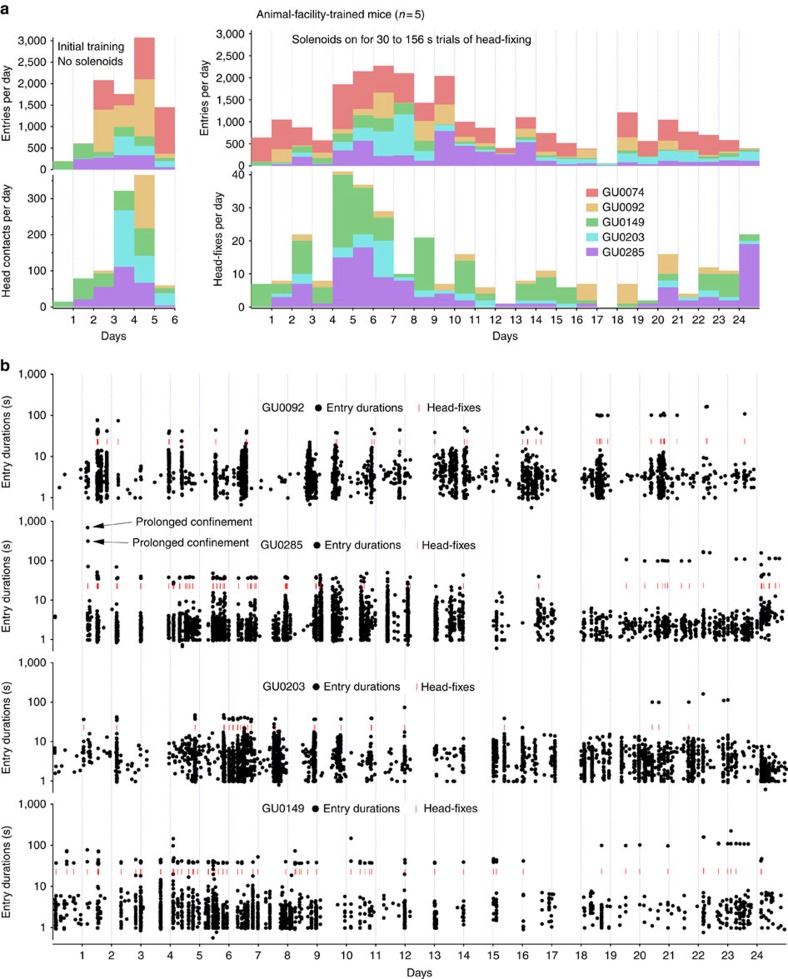
Autonomous training and initial head-fixation of facility-housed mice. (**a**) Five mice were trained within a conventional animal facility with minimal daily investigator contact. Twelve days after installation of transcranial windows, mice were started on water bottle training within a dummy cage for 7 days (no electronics, as in Supplementary Fig. 2); after this, they were switched to the head-fixing cage to receive ‘entrance' and ‘contact-based' rewards for 5 days over which time they increased entries into the fixation tube and made successful contact with the mechanism that would normally engage head-fixation. The power to solenoids was turned on and multiple head-fixes were observed within 30 min of system full function. Entries and head-fixes are plotted for the first month that this cage was functioning. (**b**) Plots showing individual entries and head-fixes (red dashes) with diurnal variation being apparent. The beginning of the plot reflects the initiation of training for three of five mice in cage GU. There were two episodes of ‘prolonged confinement', the mouse can be seen exiting the device but re-entering several more times within minutes (indicated in figure).

Five of the 16 mice that formed a single cage (designated GU) were trained 24 h per day in an unsupervised manner by placing them within the head-fixing cage within an animal facility. Facility-trained mice (similar to the other cages) progressed through water bottle training in the dummy cage over a 7-day period. The mice were then placed in the head-fixing cage (with electronics) within the animal facility. These mice were able to locate the water spout on their own within the 3D-printed head-fixation tube and received ‘entrance rewards' and ‘contact-based' rewards for 5 days before head-fixing was engaged by turning on solenoid power (Fig. 3a). Contact-based rewards are the same value as head-fixed rewards and are due to engaging the head-fixing circuitry when mice touch contact sensors with their head bars. At the end of the 5-day training period, head-fixation was activated, and, within 30 min, the first mice were head-fixed (Fig. 3b). Of five mice subjected to purely autonomous training, four were able to head-fix voluntarily. The fifth mouse was found to have residual dental cement on its bar that prevented it from being head-fixed during the initial training phase, although it still made robust numbers of entries (mouse GU0074). Our goal has been to give mice an incentive to be head-fixed (more water), but to allow them to obtain some water from entrance rewards to maintain health. Given flexibility within the software, it is possible to limit the number of entrance rewards and only dispense water for head-fixation if more sessions of head-fixation per day are required.

Head-fixation rates for the facility-trained mice were lower than some laboratory-trained animals. One notable difference was that the facility-reared mice were subject to round-the-clock trials of visual stimulation and longer trials were occasionally used (30 to 156 s). A change was made to the chamber design for the facility-reared animals only since one of 16 mice learned to enter the chamber sideways potentially allowing it to escape. An 7.5mm vertical barrier was placed at the end of the chamber to prevent sideways escape; the barrier was effective but initially lowered the head-fixing rate.

### Mechanical stability of head-fixation

**Figure 4:**
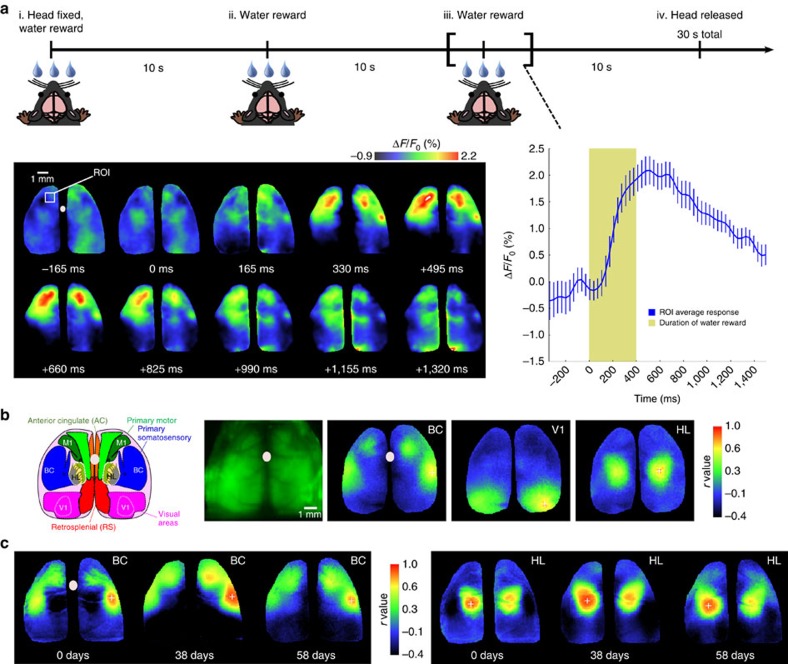
Functional imaging using auto-head-fixed mice. (**a**) Timeline for head-fixation trial with water reward delivery for Ai94 GCaMP6 mice. The mice initially get a water reward for entering the head fixation tube (valve open for 400 ms). The rewards are then given at 10-s intervals. For the third reward, the images of fluorescence from the cortex are shown. A large calcium increase is observed in the sensory and motor cortex peaking at 500 ms after reward delivery (the colour heat-map indicates change in fluorescence %Δ*F*/*F*_0_ and the white dot the bregma landmark). The plot on the right indicates the average time course for 18 trials within a single mouse (*P* value=0.0023 post-water versus baseline variation, *t*-test). The data were obtained from mouse #115 86 days after cranial window surgery. (**b**) Functional connectivity derived from imaging activity during both spontaneous activity and water reward delivery. Left: brain atlas view showing specific regions of interest in the sensory and motor cortex (adapted from the Allen Mouse Brain Connectivity Atlas). Green raw RGB image (fluorescence) of mouse cortex is shown. Far right: seed pixel-based correlation maps for barrel cortex (BC), visual cortex (V1) and hind-limb (HL) are shown. These maps are from nine concatenated trials from mouse #377 78 days from cranial window surgery. (**c**) Seed pixel correlation maps made from a single mouse spanning several months of repeated auto-head-fixation for the cortical point indicated by the plus sign (+); the heat-map indicates correlation. The maps reflect co-activated areas over nine concatenated 30 s trials obtained from mouse #115 (28, 66 and 86 days from cranial window surgery, respectively). These points for seed-pixel maps were determined on the basis of anatomical markers, such as bregma (marked by white dots) and knowledge of visually evoked map locations. Corrections for small brain movements (within trials) were implemented as described in these data sets for correlation maps only.

**Figure 5:**
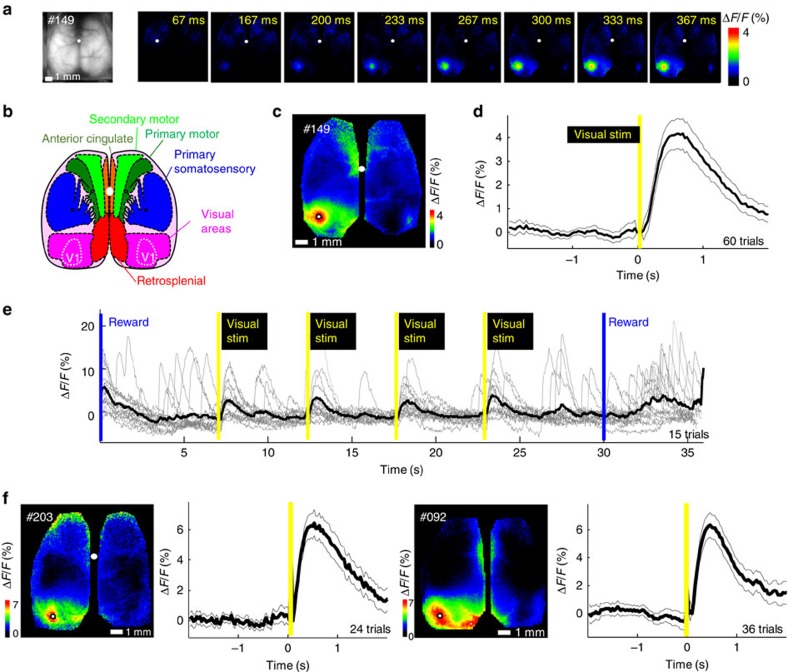
Consistent evoked visual responses observed within visual cortex of auto-head-fixed mouse cortex. (**a**) A raw epi-fluorescence image (left most) followed by a montage of averaged response (%*ΔF*/*F*_0_) following visual stimulation of the right eye (10 ms yellow LED flash placed anterior of mouse; yellow bars indicate flash stimulus) for Ai94 GCaMP6 mice from cage GU. (**b**) Atlas of the dorsal region of the cortex (adapted from the Allen Mouse Brain Connectivity Atlas). (**c**) Map of the maximum response (*ΔF*/*F*_o_). (**d**) Profile of the averaged response within a pixel located in V1 (see white dot in **c** marking maximal response) with SEM across trials indicated (mouse #0149) *ΔF*/*F*_0_ 4.9±0.6% (*P*=1.8232e−5, *t*-test, *n*=60 versus baseline variation), flash stimulus delivered at the yellow bar. (**e**) Single trials (grey lines) from the same pixel as **c** are overlayed before averaging (black line) for each visual stimulation (15 trials with four flashes each were averaged for *n*=60). (**f**) More examples of averaged responses from two other mice (mouse #0203 *ΔF*/*F*_0_ 7.6±0.8% *P*=0.00010218, *n*=24 and mouse #0092 7.3±0.8%, *P*=4.9089e−5, *n*=36), the cranial windows for these mice were made 34–40 days before the experiment. No correction for intra-trial movement was necessary in these datasets, the white dots mark the bregma landmark.

**Figure 6:**
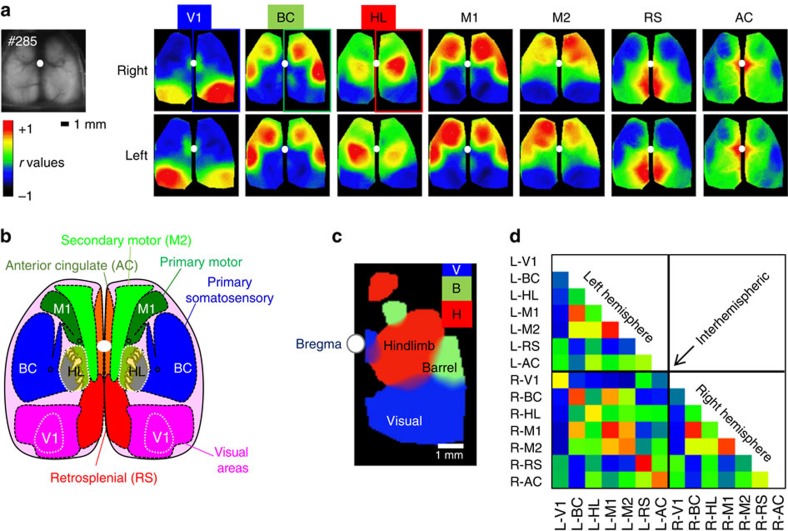
High specificity of functional connectivity mapping using resting-state spontaneous activity for mice with extended head-fixed imaging. (**a**) Spontaneous and drinking-evoked activity was collected during the extended head-fixation of GCaMP6s mouse #0285 from cage GU (total immobilized time of 1,064 s). Basal green fluorescence and correlation maps for seed pixel located in right (upper maps) and left (lower maps) V1, BC, HL, M1, M2, RS and AC. (**b**) Atlas of the dorsal region of the cortex (adapted from the Allen Mouse Brain Connectivity Atlas), much of barrel cortex is likely off the craniotomy. (**c**) Composite thresholded map of the high correlation region showing the remote ipsilateral connectivity for seed pixel in V (blue, visual), B (green, barrel cortex) and H (red, hindlimb). Islands of remote co-activation are present in the motor cortex for HL and BC seeds (smaller red and green blobs, respectively), while V1 seeds show remote activity in AC near the white dot marking bregma. (**d**) Matrix of correlation between regions presented in **a**. No correction for intra-trial movement was necessary in these data sets. The window was installed 25 days before acquisition.

We assessed mechanical stability of head-fixation by examining the relative *x*/*y* position of landmarks such as blood vessels both within and between sessions of head-fixation. Between 30-s sessions of head-fixing, such landmarks drifted about one pixel (45±4.5 μm; 257 head-fixes in five mice; Supplementary Fig. 3A). Supplementary Video 3 shows raw unprocessed or aligned GCAMP6 fluorescence data and illustrates the stability. Small translations between 30-s imaging sessions were corrected in software during concatenation of files from different head-fixed sessions. In addition to *x*/*y* displacement, we also examined *z* axis stability within a 30-s session using a laser range finder (Keyence LK-G; placed at approximate centre of window). For well-trained mice, vertical displacements within a trial had a deviation of 9.5 μm (RMS based on 27 head-fixed sessions). Although these movements were larger than previous applications where two-photon imaging was performed^17^, they were still relatively small compared with the estimated depth of field of >1 mm (see ‘Methods' section) and pixel size of 42 μm. Importantly, assessment of GCaMP6 mice indicated no correlation between Z-drift and *ΔF*/*F*_0_ signal (see Supplementary Fig. 3B,C). To assess the brain activity, we collected sequences of green epi-fluorescence images using the Raspberry Pi camera. A simple epi-fluorescence system was used with an LED light source (with excitation 475/30m and emission filter ET525/36m Chroma) similar to what we have previously developed^3^,^12^. We stress that images of mesoscopic activity are well suited for this form of self-head-fixing since they are less subject to the effects of movement artifacts given the lower resolution and large focal volume (depth of field). The goal of this activity-dependent imaging is to assess mesoscale maps (over up to millimetre scales), and not to resolve individual cells or responses within particular layers of cortex, although possible with other measures^19^. The large focal volume is expected to be more forgiving with regard to variability in the brain surface due to curvature. The images were processed as previously reported^3^,^12^ using procedures written in Python and in some cases Matlab (see Supplementary Fig. 4). Briefly, sequences were aligned using automated procedures between sessions of head-fixation and band-pass filtered at 0.3–3 Hz. Fractional change in fluorescence (*ΔF*/*F*_0_) was calculated and global signal fluctuations (over the entire brain window) were regressed to remove potential global sources of noise and to heighten the contribution of local networks^12^. Using these procedures, little signal was observed in animals without GCAMP6, but expressing fluorescent GFP (Supplementary Figs 5,6 and 7). In some cases (correlation maps in Fig. 4b and Supplementary Fig. 6B only) we corrected for small shifts in intratrial focus by using areas in the field of view outside the transcranial window. For example, a correction region of interest (ROI) was chosen in dental cement or in a location to which we added fluorescent paint. The fluorescent paint served to enhance the signal from the correction ROI and improve the focus correction. As fluorescence changes in the correction ROI were mainly attributable to motion, division by the average time course of the correction ROI reduces the contribution of focus shifts to the measured *ΔF*/*F*_0_. These corrections were only implemented if detectable movement-related signals were obtained from the correction ROI and the correction-reduced RMS signal in areas outside the transcranial window. These corrections were not used in the refined versions of the cages where stiffer solenoid rods were used (Figs 5 and 6 and Supplementary Fig. 8). Supplementary Video 4 shows a processed GCaMP6s recording (temporal filtering, global signal regression and *ΔF*/*F*_0_ calculation, but no intratrial movement correction), which can be compared with Supplementary Video 3, which is unprocessed.

### Mesoscale cortical mapping in automatically head-fixed mice

We were able to define robust maps associated with the somatosensory and motor system while animals were waiting for water, drinking and processing stimuli when head-fixed. Expressing results as a %*ΔF*/*F*_0_, we observed robust activation within sensory and motor areas that peaked within 500 ms of water delivery (Fig. 4), these reward signals were not observed in GFP-m mice (Supplementary Figs 6 and 7). The activated regions during drinking included the anterior medial and lateral motor cortex that have been implicated in other water reward tasks^15^; see Supplementary Video 5.

Using seed-pixel-based correlation analysis^3^,^12^, based on activity during 30-s head-fixed session that included water reward, we found that seeds placed in barrel cortex resulted in expected long-distance functional connectivity to motor areas^3^,^7^,^12^. The general pattern of activity also agreed between different mice (compare Fig. 4b,c), and was consistent with previous work^12^. GCAMP6 expression in these animals is dependent on a CaM kinase promotor-driven expression system so signals are expected to be largely attributed to activity within neurons^10^.

The same animals could be repeatedly mapped in different sessions with brains being registered to within micrometres (corrected in software; Fig. 4c). Over the course of daily imaging for a 2-month period, we observed similar maps made using correlation over time (Fig. 4c). Furthermore, both sensation-evoked and spontaneous activity mapping was feasible in windows 60 days after implantation (see Fig. 4 and Supplementary Figs 5 and 7). It was not necessary to align images within 30-s epochs collected within a single session of head-fixation after discarding the first 0.3–3 s of data that contained movement associated with mechanical settling after the fixation itself (see Supplementary Video 3 for raw example). Image stacks were aligned between sessions of fixation.

Assessment of correlation-based maps in GFP-m animals indicated little connectivity for V1 and BC seed pixels. However, we did observe a significantly smaller degree of correlated activity in the hindlimb area (*r*=0.3) when using GFP mice (Supplementary Fig. 6B). It is likely that this signal results from very strong connectivity within the hindlimb area and interactions with hemodynamic effects and GFP green epi-fluorescence, since reasonable functional connectivity can be observed using imaging of spontaneous intrinsic optical signals^12^,^19^,^20^. The hindlimb map is relatively symmetrical, so even a small local correlated signal will resemble general features of this map particularly as correlation values are independent of signal amplitude. We anticipate that as red-–shifted calcium and voltage indicators are further optimized^5^,^10^,^21^,^22^, these other signals will have an even smaller contribution. Importantly, light-activated signals were not observed in GFP-m mice under the conditions used (Supplementary Fig. 7).

### Assessment of cortical visual responses

Using Ai-94 GCAMP6s mice that were maintained within an animal facility, we assessed visual responses in unsupervised experiments. We used 10–20 ms yellow light flashes that were directed predominantly to a single eye and placed in the anterior visual field at an elevation of 0 degrees (Fig. 5, data from three mice #0149, 0092 and 0285 are shown; four flashes 5 s apart). The flashes were delivered randomly in 65% of head-fixed trials that lasted from 35 s (four flashes 5 s apart) or in some cases as indicated 95–150 s trials with 12–20 flashes 5 s apart (Supplementary Fig. 8). GCAMP6 signals were initiated in the location of visual cortex ∼4.5 mm posterior of bregma and spread medially and were associated with secondary activation in cingulate cortex consistent with previous work^3^.

In earlier experiments, we used brief light flashes (390 nm) directed in the lateral visual field and placed near each eye. The ultraviolet flashes were well perceived by mouse retina, while well separated from the imaged green epifluorescence and have little interference with GCaMP fluorescence measurements. These flashes led to lateralized visual cortex GCaMP responses (in three separate mice; #0115, 0159 and 0377) that were distinct from slow residual global movement or hemodynamic artifacts that are only expected to be present in GFP-m control mice (#0300 and 0245; Supplementary Fig. 7; ref. 23).

Previous work from our group^12^ and others^6^ concluded that under the conditions we use for imaging hemodynamic signals have little interference with GCaMP fluorescence. This was confirmed in two control animals that expressed GFP, but not a calcium sensitive probe for water reward and ultraviolet flash stimulation (Supplementary Figs 6 and 7, respectively). Although hemodynamic signals will affect fluorescence, the larger *ΔF*/*F*_0_ of GCaMP6 leads to a smaller relative contribution and makes small auto-fluorescence contributions from flavoproteins negligible^24^.

### Optimizing trial duration

Trials for automatic head-fixation were initially set at 30 s duration during the first weeks of training since previous rat studies using both self-directed fixation and release indicated that durations as short as 8 s were chosen by rats themselves^17^. We used 30 s as a compromise duration and an acquisition time that was capable of making a functional connectivity map from spontaneous cortical activity data^12^. Given that there are multiple animals within a cage, we were reluctant to initially increase duration to 10s of minutes since head-fixed mice could be vulnerable to aggression by cage-mates as they compete for a source of water. However, this issue is less significant for female mice that are housed 24 h per day in the head-fixation cage with water continuously available from the apparatus. We believe that mouse home-cage head-fixation can be extended in trial duration as in published abstract form^25^ to accommodate more complex behavioural imaging or optogenetic procedures that begin to mirror session lengths typically used with manually head-fixed mice^15^. The mice were able to tolerate increasing trial length (Fig. 6 and Supplementary Fig. 8) and rewards can be placed at the beginning and end of the trial to allow more time for experiments in between (see Fig. 5 for example of trial structure). Relatively short intervals between repeat head-fixations was still observed when the trial length was increased from 30 to 64 s (82±136 s for 30-s trials and 122±177 s for 60-s trials, *P*=0.055 unpaired *t*-test, 771 and 78 head-fixes, respectively) indicating that 64 s is non-aversive. Furthermore, in visual stimulation trials, high-quality data were obtained in trials lasting 97–156 s where 12–20 flash stimuli were delivered randomly to head-fixation trials (Supplementary Fig. 8 for 20-flash example).

In one case, we unintentionally induced longer periods of confinement lasting 11.5 and 5 min. Neither of these episodes led to the harm of any mouse and they were able to exit the device without assistance. Longer periods of data acquisition revealed more subtle intra-hemispheric functional connectivity maps and interactions between visual and cingulate, barrel and motor, or limb region of the somatosensorimotor and anterior motor regions (blue, green and red, respectively, Fig. 6 and Supplementary Video 6). Correlated activity (reflecting functional connectivity) within the retrosplenial, anterior cingulate and motor areas for both hemispheres was observed for seed pixels placed in these areas (Fig. 6). In the case of the visual cortex, we show an example of remote correlated activity in the anterior cingulate cortex (Fig. 6) with a V1 seed pixel reproducing findings observed with voltage-sensitive dyes^3^.

Anecdotally, mouse #285 in Fig. 6 was head-fixed for 11.5 min and was released, then re-entered the tube 12 times within the next 23 min and was head-fixed two additional times (Fig. 3b, marked prolonged confinement). Prompt re-entry of mouse #285 suggested that 11.5 min of confinement was not overtly aversive. On the basis of these preliminary findings, we anticipate that sessions of head-fixation and imaging could be significantly extended to a 10-min period to collect data during an automated behavioural assessment consistent with recent work published in abstract form for mice^25^.

## Discussion

We have described a high-throughput system for the autonomous collection of self-initiated imaging of spontaneous and sensory-evoked activity. This setup and training philosophy could also be applied to chronic multisite photostimulation^15^,^26^ of cortex using Channelrhodopsin-2 or other opsins^21^, potentially allowing photostimulation to be dependent on local activity and delivered in a closed-loop manner^27^. We also anticipate extension to imaging changes in blood flow or other metabolic signals^24^,^28^,^29^ and using strategies to image-depth-resolved activity over wide scales^30^,^31^ that could include other imaging strategies such as ultrasound^32^ or optical coherence tomography^33^ that operates over similar spatial scales. In preliminary work, we find that the red channel of the RGB image is sufficient to selectively resolve simultaneous green epifluorescence and red intrinsic signals.

The design of our system also supports additional behavioural tasks, and even changing them remotely through the web secure shell interface. The benefits of automated, home-cage assessments include reduction of experimenter bias^34^ and physiological stress response induced by handling^35^. In addition, providing access to water 24 h per day (by engaging in the task) decreases the potential detrimental effects of prolonged water deprivation protocols^36^ where the entire daily water quota is provided at once. Several home-cage tasks for assessing both cognitive^37^ and motor behaviours^38^ are currently available; however, simultaneous imaging of brain activity has not been previously performed.

We have collected images during the presentation of water rewards, which does result in a significant amount of activation in sensory–motor areas expected to be associated with licking and drinking^15^. We note that the task is flexible and in some cases we have withheld rewards until after a period of image acquisition allowing a more faithful assessment of spontaneous (non-task-related activity), or activity associated with sensory cues such as light flashes. Recent work with auto-head-fixing rats indicates that high trial numbers (500 head-fixes per day) support studies of visual system function where internal (visual cortex) estimates of visual stimuli can be compared with numbers of stimuli delivered within a task^18^. Although, in our work, mice head-fix at considerably lower rates than rats, there is potential to create longer trials where mice are only head-fixed a few times a day but for 10s of minutes after determining that such procedures are not stressful. Consistent with our observations, is work published in abstract form^25^ where sophisticated behavioural tasks were used in mice that were automatically head-fixed for 30-min periods, but for only once a day. However, this work did not explore the feasibility of brain imaging or tracking multiple mice within the environment of an animal facility.

In conclusion, the self-contained nature of these home cages permits them to remain in an animal facility preserving colony integrity and facilitating high-throughput cortical physiology under defined conditions. We also anticipate that the cages will enable physiological assessment of animals with altered microbiomes, or exposure to dangerous infectious agents and circadian rhythms in a less-invasive manner.

## Methods

### Animals

The female transgenic C57BL/6 mice expressing GCaMP6s/f (Ai94 or Ai93, from Allen Institute for Brain Science, crossed to Emx1-cre and CaMK2-tTA line, Jackson Labs) and GFP-m (Jackson Labs)^23^ were used in this work and underwent surgery, water restriction and training is described below. The average age of mice at the time of the first procedure (cranial window surgery) was 79±25 days. All the procedures were conducted with approval from the University of British Columbia Animal Care Committee and in accordance with guidelines set forth by the Canadian Council for Animal Care. The mice were housed in a conventional facility in plastic cages with micro-isolator tops and kept a normal 12-h light cycle with lights on at 0700, h. When the mice were placed in auto-head-fixing cages, the bedding was removed and substituted by paper towels to prevent the fine pieces of bedding interfering with the head-fixing apparatus. The lack of bedding was a precaution; we anticipate that it would be possible to use bedding in the future.

### Animal surgery

The animals were anaesthetized with isoflurane and a through-bone transcranial window was installed as previously described^12^,^39^,^40^. Briefly, a skin incision was made and the skin over cortex was retracted and glass coverslip was applied using Metabond clear dental cement (Parkell, Edgewood, NY, USA; Product: C&B Metabond). The coverslip was applied to un-thinned bone that was coated with Metabond. During the same surgery, the steel fixation bar was placed so that there was a 4-mm posterior space between the bar edge and bregma. A small incision was made over the neck and an RFID tag (125 kHz glass Sparkfun) was implanted and secured by a suture.

### Camera parameters

To focus the image at ∼15 mm from the sample, the Picam lens was screwed to its maximal counterclockwise position (threads engaged about half turn). The depth of field was determined in the same way as in previous reports^41^ and was found to be >1 mm. This provided both a large focal volume over which to collect fluorescence and makes the system less sensitive to changes in *z* axis position of several 100 μm. The recordings were saved in H264 compressed mp4 files or raw 24-bit RGB. To reduce the image file size, we binned data at 256 × 256 pixels on the camera. To collect the consistent images, we manually fixed camera frame rate to 30 Hz, turned off automatic exposure and auto white balance and set white balance gains to unity. Other parameters for imaging (blue light excitation and green emission) were similar to previous work using GCAMP3 (ref. 12). To register frames within and between recording sessions, the images were aligned using shifts computed by cross-correlation, which was calculated directly (MATLAB) or via the FFT to estimate shifts (Python).

### Automated head-fixation setup

The head-fixing device was designed around electromagnetic solenoid-driven pins, which provide reproducible fixation of mice with head bars. The initial design used Planetengineers.com push-type solenoid (S-15-75-28-H). Rigidity of the head-fixation was improved using larger solenoids: 8 mm 24 V push solenoids (Aliexpress 2026619226). The solenoids were positioned at a 30-degree angle to the head bar and small angular adjustments (<2 degrees) were made during setup to ensure strong fixation and prevent binding on release. As the maximum force from the solenoid occurs near the end of its stroke, the solenoid position was adjusted such that the final position of the end of the pin was 1–2 mm past the contact point with the head bar. The system was tested a minimum of 30 times with a dummy head bar mounted on a steel rod before being tested on animals following any modifications. During repeated fixation, the solenoids will generate heat and, as a precaution, we added heat sinks and small fans to keep the solenoids cool. This mitigated any potential heat transfer to the head bar. Two modifications were made to the solenoids to improve fixation and release. The supplied 3-mm pins were replaced with larger rods made of one-eighth of an inch tool steel with rounded ends and an additional spring was added to the two supplied to increase the restoring force. The springs ensure the system would release mice in the event of a power failure or other unforeseen glitch.

### Training of mice for self-head-fixation

The female mice that were on average 79±25 days of age and had an initial weight of at least 18 g were used. All the mice were at least 2 months of age before any water restriction was begun. We performed a surgery to install a chronic transcranial window and head fixation bar^12^,^39^ that was similar to windows with bone thinning^11^. Five to 12 days after the surgery, the animals were placed on a schedule of water restriction and had access to a training cage. Given the variation in weight due to *ad libitum* consumption, the initial mouse weight was defined 24 h after the start of water restriction. From this weight, mice could lose up to 15% of body weight (before being supplemented), but then regained much of it once they became proficient at the head-fixing and water delivery task. After more than 2 months of daily auto-head-fixing female mice averaged 21.2±2.2 g (*n*=11 mice), which is within 1 s.d. of the normal growth curve for C57bl6 female mice (after accounting for the weight of the head-bar). Animals were restricted to a one ml per day water ration during initial laboratory training and were supplemented if there were any signs of distress or excessive weight loss, as previously reported by Svoboda and colleagues, or failed to obtain water through sufficient head-fixed or entrance rewards^15^. If mice did not progress well through training they were still given 1 ml of water daily usually within the head-fixing tube (water pipetted on floor to facilitate access). Once mice have learned the head-fixed task, we allowed them to automatically head-fix themselves and consume water *ad libitum*. Successfully head-fixing animals were able to maintain body weight and gained weight towards pre-surgery and water restriction values. To train new animals, water-restricted mice (1 ml per day) were exposed for 5–7 days to the automatic head-fixation cage (or a dummy system) where they received water rewards without head fixation (see Supplementary Fig. 2). Both intermittent (∼4 h periods each day) and 24 h per day home cage training was effective for training naive animals. Within these dummy cages that lacked electronics, the mice learned to associate the head-fixation tube with water and drank water pipetted within the tube entrance, or from a water bottle spout which was progressively moved to the position where head-fixation normally occurred (Supplementary Fig. 2). Over the 7–10-day period, the mice progressed further into the tube (by moving the water spout more distally within the tube). The mice were then switched to the automated system where they received computer controlled water rewards for entering (∼5-8 μl drops), but no head-fixation. The spout position was then moved further back until it reached an optimal position for head-fixation. Importantly, mice still had access to some water (5–8 μl entrance rewards) by extending their paws (or tongue) to grab water drops at the end of the spout without engaging head-fixation. However, if head-fixed, they would receive ∼4–30 times water and have the chance to remain fixed and obtain a second set of drops during a subsequent trial. Once all the animals were making up to hundreds of tube entries per day (durations between 1 and 20 s), the spout length was further adjusted to make it more likely that they would be head-fixed (but not required to obtain water). Within 7 days of the initiation of water restriction, the mice were beginning to become automatically head-fixed. By 14 days from the start of water deprivation, 15 of 16 mice studied were repeatedly head-fixing themselves. The procedure was not thought to be aversive since some mice would repeatedly head-fix themselves by voluntarily staying within the apparatus and this was evident by observing repeat fixations within less than 1 min (see Fig. 2c and see Supplementary Video 2 for an example). In most cases, pure water was dispensed (cages EP, EP and AB); in one cage (GU), water with 10% sucrose was used to stimulate initial training (for 10 days), but this was subsequently discontinued due to little noticeable advantage. Although not used in the data sets presented in this paper, two modifications of the training procedure were subsequently made. During the training phase when mice engage head-fixing circuitry (but are not head-fixed), a dummy solenoid (attached to the side of the cage) was used to simulate the sound and vibration of the process with the aim of habituating the mice to solenoid sound. Probabilistic head-fixing was also implemented where mice were head-fixed in only 65% of randomly selected trials throughout the training and assessment process. Non-head-fixed trials still dispensed water rewards of the maximum value.

### Training timeline

Training can take place daily in the laboratory, or autonomously within an animal facility. Training procedures are outlined below. Week 1–2: install coverslip window and attachment bar, allow 5–10 days of recovery from surgery *ad lib* water in regular cage. Week 2–3: begin by restricting the mice of water for 24 h, establish weight cutoff of 15% max loss for each mouse. Give *ad lib* water given in ‘dummy' head-fixing cage (Supplementary Fig. 2). Over 6–9 days require mice to access water bottle in fixation tube (no head-fixing initiated or limits on water consumption), progressively move water bottle to distant holes confirming consumption by bottle weight change. Weigh mice every 1–2 days and supplement water if they do not obtain 1 ml per day or meet weight targets. Week 3: place mice in auto-head-fixing cage and allow high limit of water drops for tube entry (500 entrance rewards), position lick spout so that all mice can obtain water with or without head-fixing. Give significantly more water for initially contacting sensor plates (first 5 days) or head-fixing. Use 1-4 h training sessions in lab or continuous 24 h per day training in the animal facility. Weigh mice every day to ensure all get at least 1 ml a day or supplement. Week 4–5: progressively move water spout into position where the mice are more likely to be head-fixed, place no limits on number of head-fixed water rewards. Limit entrance rewards to 100–350 and use 1–4 h laboratory or 24 h per day training. Weigh mice every 1–2 days, ensure that all the mice get 1 ml per day or supplement water.

### Raspberry Pi and electronics interface

To control the task, we used the Raspberry Pi 2 single-board computer. This internet-accessible device incorporates HDMI video output and was able to both control the task and capture the data. To collect the images of mouse entry and exit from the cage, a second Raspberry Pi camera was used. See Supplementary Fig. 1 for a block diagram of the hardware. Assigning each Raspberry Pi a unique IP address allowed us to assess the cages remotely through the internet to monitor whether any animals were in difficulty during chronic imaging or not receiving sufficient water rewards.

### Statistics

The results are presented as mean±s.d. unless noted otherwise. Experimenters were not blinded during the experiment or the analysis, although automated acquisition and analysis procedures were used. No animals were excluded from the results and no method of randomization was used. The sample size measurements for the analysis of imaging data were consistent with previous work^3^,^12^. To test the hypothesis of visual response, paired *t*-tests were performed between maximum Δ*F*/*F*_o_ responses processed after and before visual stimulation. Two-tailed unpaired *t*-tests were used to assess significance otherwise.

### Code availability

Example code to run the home-cage system is available at: http://www.neuroscience.ubc.ca/faculty/murphy_software.html

A file to 3D print the head-fixing tube is also available there (printed using Form1+ from Formlabs).

### Data availability

The data that support the findings of this study are available from the corresponding authors upon request.

## Additional information

**How to cite this article:** Murphy, T. H. *et al.* High-throughput automated home-cage mesoscopic functional imaging of mouse cortex. *Nat. Commun.* 7:11611 doi: 10.1038/ncomms11611 (2016).

## Supplementary Material

Supplementary Information Supplementary Figures 1-8

Supplementary Video 1 Overhead view of group housed, transgenic mice with steel head fixation bars and transcranial windows.

Supplementary Video 2 View of a mouse being repeatedly head-fixed at the end of the head fixation chamber.

Supplementary Video 3 Raw GCaMP imaging data from an automatically head-fixed mouse.

Supplementary Video 4 Fractional change in fluorescence recording for single trial of automatic head-fixation.

Supplementary Video 5 Average fractional change in fluorescence over 18 automatic head-fixations with water reward delivery.

Supplementary Video 6 Montage of correlation maps for seed pixel travelling through right M1, HL, BC, V1 (monocular and binocular) and then RS.

## Figures and Tables

**Table 1:** Parts list for the main components to build the automated home page for self-head-fixation and imaging.

**Component**	**#**	**Vendor**	**Product ID**
Raspberry Pi Model B 2	1	Newark	38Y6467
8gb SD card	1	Adafruit	1583
Powered USB hub for hard disk	1	Staples	22316
USB hard disk 2TB	1	Seagate	STDR2000100
Raspberry Pi Dish	1	Adafruit	942
L293D	1	Adafruit	807
L298N	1	Amazon	B00AJGM37I
Pi Cobbler +	1	Adafruit	1989
Water valve solenoid	1	Newark	45M6131
Linear lift electromagnet Solenoid	2	Aliexpress	2026619226
Solenoid (initial design only)	2	Planetengineers	S-15-75-28-H
RFID Reader ID-12LA (125 kHz)	1	Sparkfun	11827
SparkFun RFID reader breakout	1	Sparkfun	13030
RFID glass capsule (125 kHz)	# of mice	Sparkfun	9416
RPi camera (F)	1	WVShare	RPi camera (F)
Chromoly steel (head bar)	1	McMaster-Carr	4468T11
ET525/36m 10 mm filter	1	Chroma	ET525/36m

With the exception of the fluorescence filters that could be substituted with cheaper coloured glass variety, the main components are less than $300 per cage. Example code to run the home-cage system is available at: http://www.neuroscience.ubc.ca/faculty/murphy_software.html. Stl file for 3D printing the head-fixation chamber (printed using Form1+ from Formlabs) is provided at the same location. LED fluorescence excitation was as described previously^12^.

**Table 2:** Head-fixing rates for all mice studied.

**Animal ID**	**Entries**	**Head-fixes**	**Days tested**	**Entries per day**	**Headfixes per day**	**Image data**
AB0090	9,944	145	90	110.5	1.6	
AB0573	5,335	143	90	59.3	1.6	
AB0592	5738	233	90	63.8	2.6	
EL0115	8,772	1,072	90	97.5	11.9	Fig. 4
EL0159	8,879	619	90	98.7	6.9	Supplementary Fig. 5
EL0245	11,195	425	90	124.4	4.7	Supplementary Fig. 5
EL0300	6,345	1,076	90	70.5	12.0	Supplementary Figs 5 and 6
EL0377	7,018	2,021	90	78.0	22.5	Fig. 4
EP0139	7,742	178	90	86.0	2.0	
EP0202	4,237	932	90	47.1	10.4	
EP0238	5,636	176	90	62.6	2.0	
GU0074	10,512	0	25	420.5	0	
GU0092	3,525	39	25	141	1.6	Fig. 5
GU0149	2,191	133	25	87.6	5.3	Fig. 5
GU0203	4,173	40	25	166.9	1.6	Fig. 5
GU0285	5,036	107	25	201.4	4.3	Fig. 6

Cages AB, EL and EP were laboratory trained, while cage GU as trained in an animal facility.
