# Supplementary material for: High-throughput automated home-cage mesoscopic functional imaging of mouse cortex
Source: Nat Commun. 2016 Jun 13;7:11611. doi: 10.1038/ncomms11611 (PMC4909937; doi:10.1038/ncomms11611)
Supplement: Supplementary Information — Supplementary Figures 1-8 [file ncomms11611-s1.pdf]

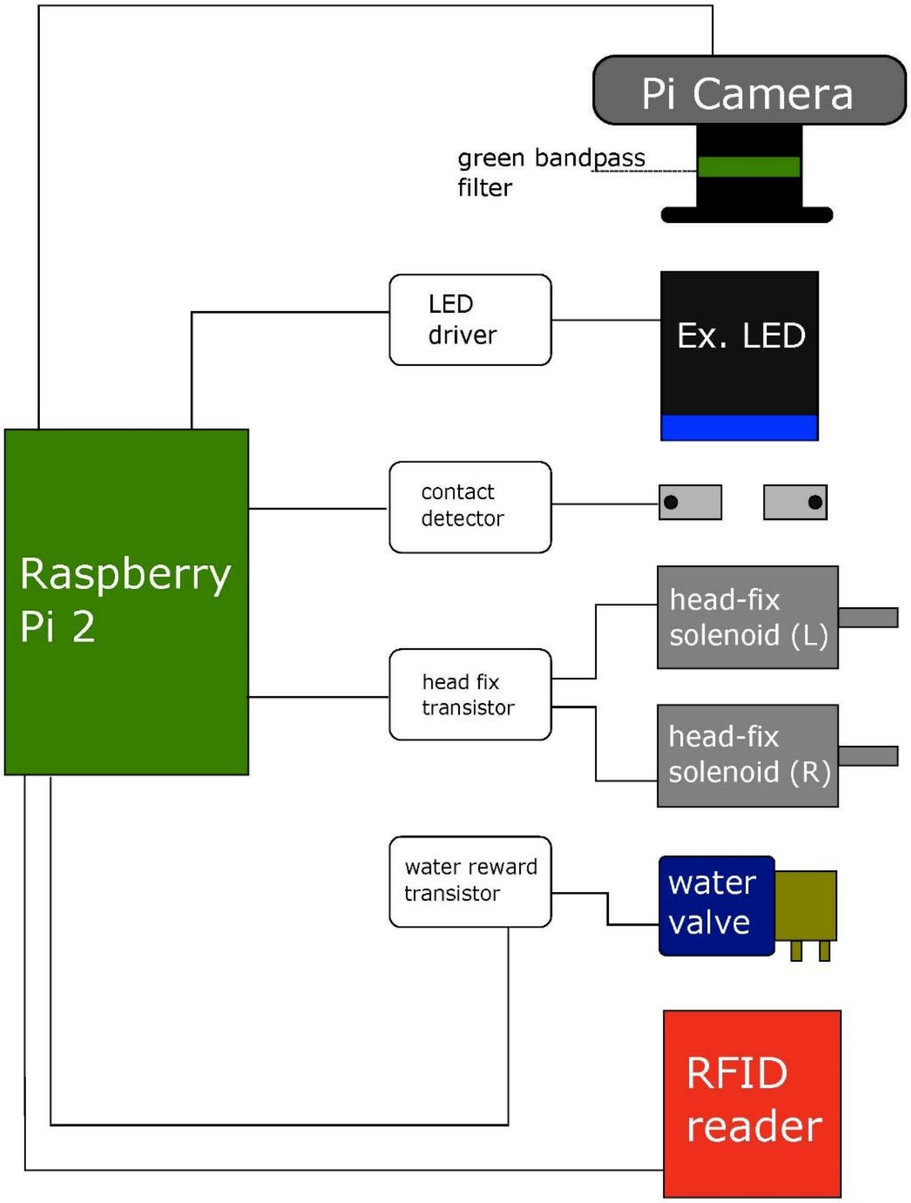

Supplementary Figure 1. Diagram showing software and hardware used for head fixation and water delivery, as well as picam-based imaging.

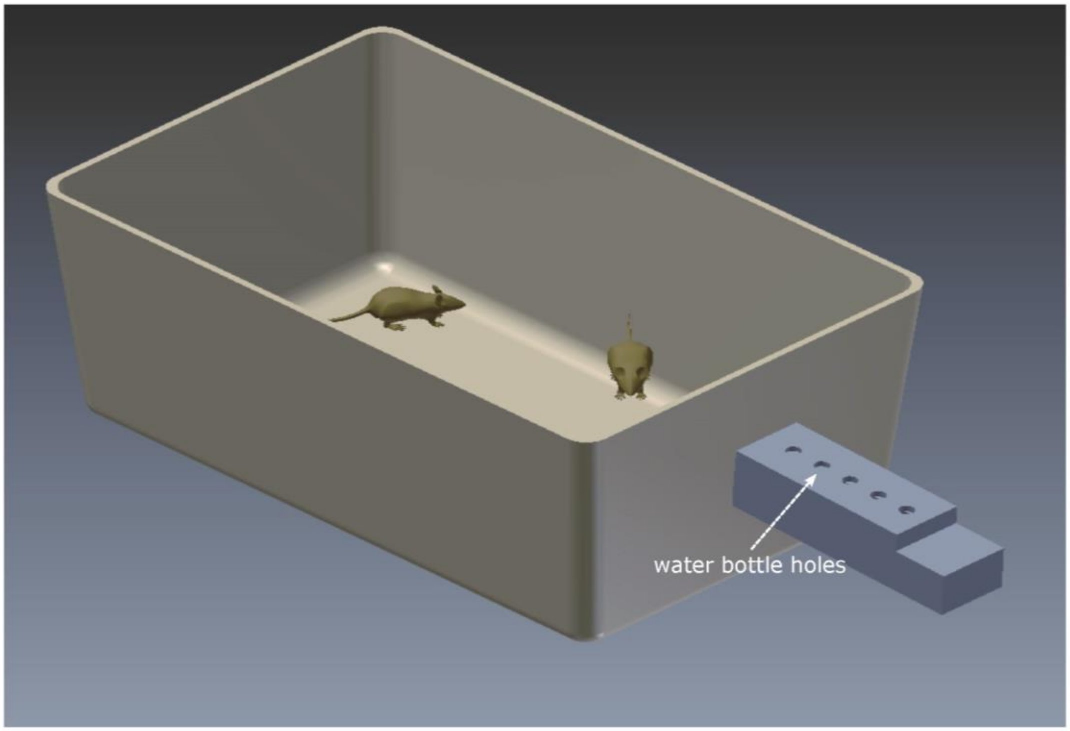

Supplementary Figure 2. Layout of training cage for self-head-fixation. These dummy cages contained no electronics and were used for daily water supplementation (if animals were not at target weight) and for initial training for head-fixation. During training, we confirmed that mice were drinking from more distal water bottle positions by daily weighing of the bottle. The opening to the chamber was identical to the version of the cage used for mechanical head-fixation. A small water bottle was made from a 50 ml conical tube and a straight ball bearing-containing spout (8 mm diameter tube) that protruded 10 mm below the roof of the fixation tube near the level of the eventual water spout.

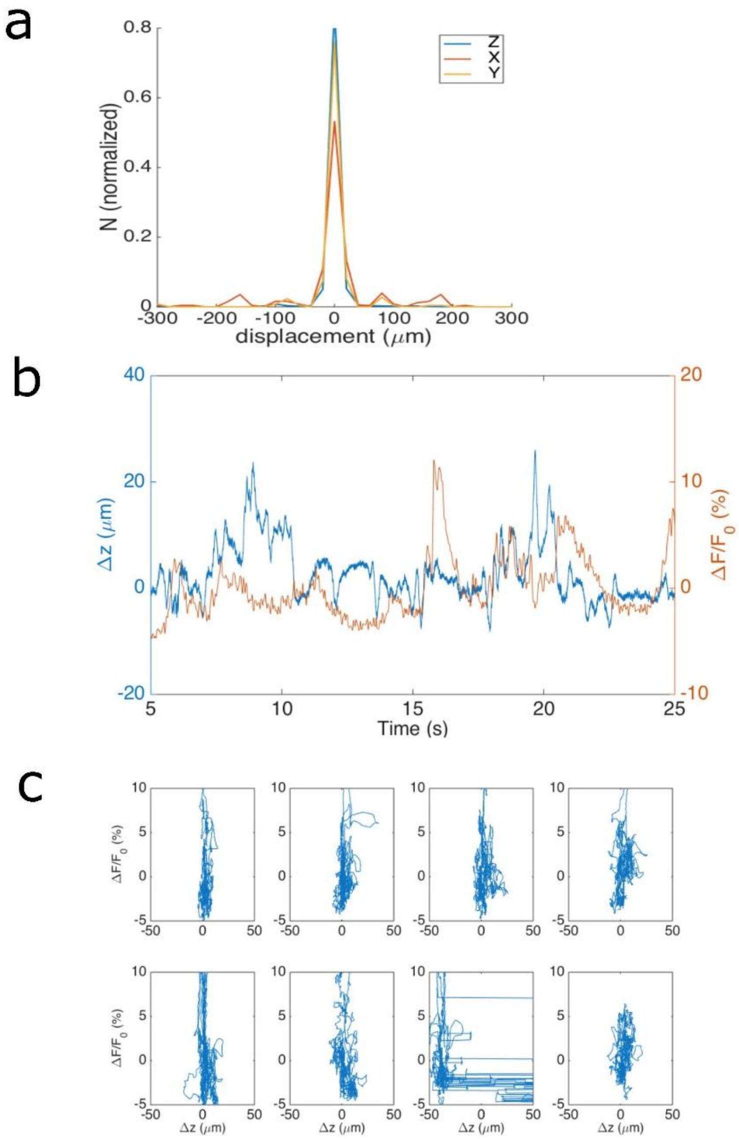

Supplementary figure 3. Correlation between Z axis movement and dF/Fo.
a) X,Y,Z assessment of stability of head-fixation normalized values. X and Y values represent between trial variation while the Z axis represents movement within a 30 s imaging session.
b) No apparent relationship between Z drift and dF/Fo signals. Using GCaMP6s transgenic mice, we plotted dF/Fo values during spontaneous activity and water reward delivery. The overplots indicated no relationship between Z position measured with laser range finder and dF/Fo. c) Plot indicating Z position and dF/Fo signals for various points during data acquisition showing little significant correlation. We have included a trial where there was an abnormally high z-axis fluctuation and still no apparent correlation between the change in z-axis position and dF/Fo signal was observed (3rd plot in C). The range finder was positioned in the center of the transcranial window.

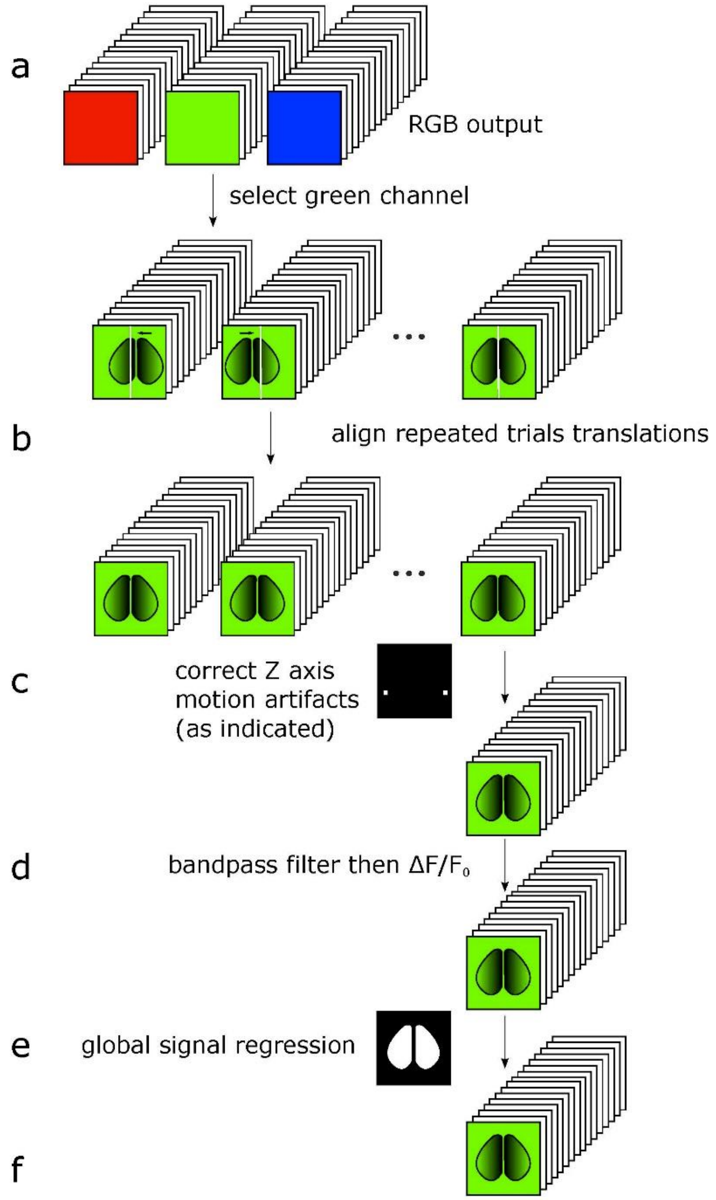

Supplementary Figure 4 Schematic of imaging data analysis pipeline using python. a) Raw data collected as 24 bit RGB frames at 30fps, we select only the green frames. b) Using an image registration library for python we correct any shifts on the X and Y axes to ensure all the trials are aligned (X, Y alignment was only done between trials and not within trials). c) For correlation maps only as indicated below, we decreased the signal contribution of Z axis movement artifacts by assessing signal from fluorescence located within non-brain regions found at the outer edge of window near the middle of the anterior-posterior axis and divided by a proportionally scaled version of this signal (Fig. 4b,c and Supplementary Figure 6b). d) We filter the frames using a zero-phase lag Chebyshev bandpass filter at 0.3-3 Hz and we calculated the fractional change in fluorescence (dF/Fo) on the resulting signal. e) Finally, the global signal in the frames was regressed. f) The resulting processed data can be concatenated to create the seed pixel correlation maps or can be averaged to create an evoked activity trial.

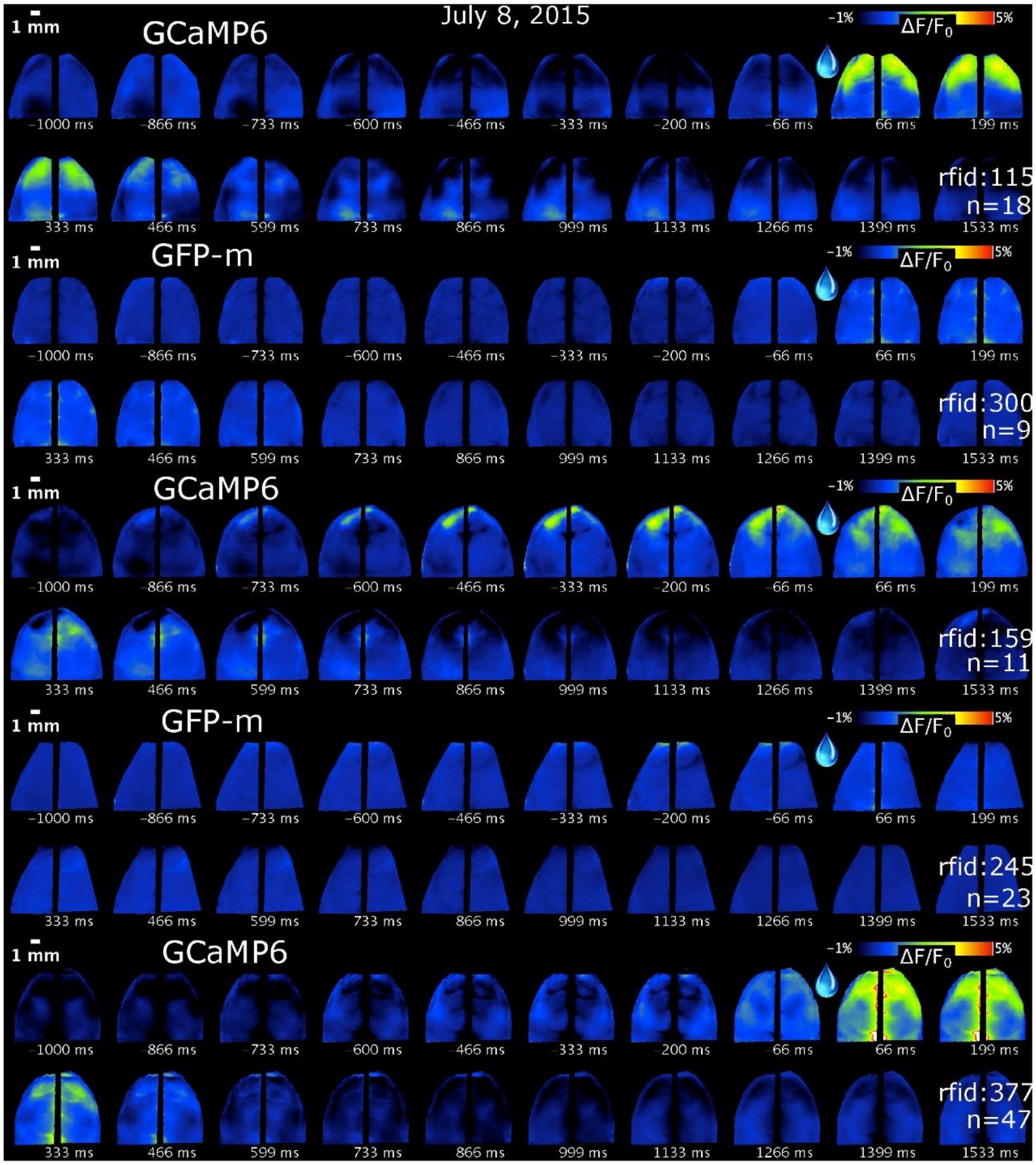

Figure 5. Water reward brain activity montages. Changes in brain fluorescence elicited by a water reward for three GCaMP6s (#0115, 0159, 0377) and two GFP (0245, 0300) mice. A strong signal is observed in anterior sensory and motor areas after the reward is given to GCaMP6s mice. Little to no changes were observed in the GFP-m mice. The montages span a total of 2.5 s of brain activity for each of the 5 mice and are an average of at least 9 trials. The data was obtained 60 days after the cranial window surgery. Data from mouse 0159 shows some activity in anterior motor areas prior to reward that may be related to anticipatory licking.

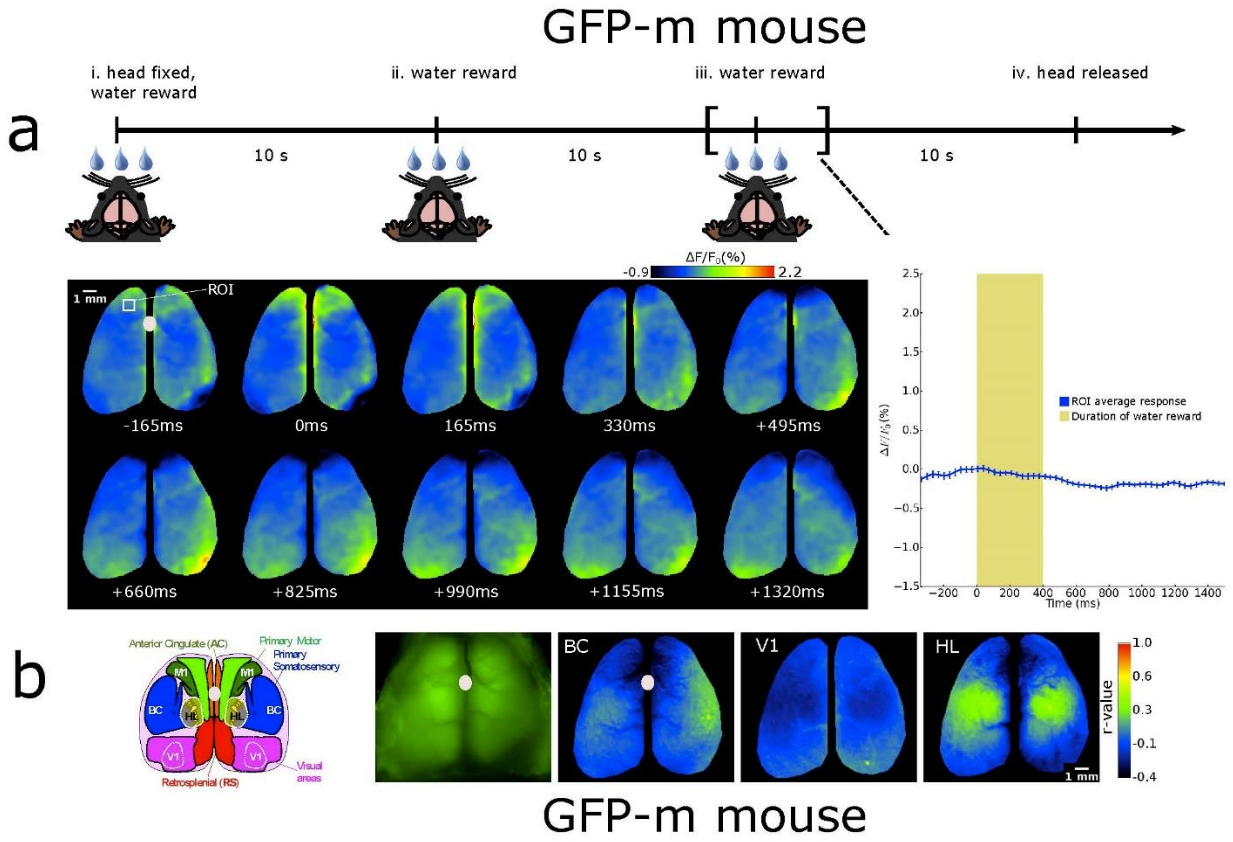

Supplementary Figure 6. Water reward and correlation maps made from control GFP-m mice. a.) Little water and drinking-evoked fluorescence signal was observed in GFP-m mice (p-value=0.09 versus baseline, example from mouse #0300). Error bars indicate the SEM across 26 trials. **b.)** Correlation maps made during 30 s epochs of water rewards also showed little structure when compared to GCaMP6, although the hind-limb area did show lower amplitude (by correlation) activity that was potentially associated with residual hemodynamic responses in this area that typically shows very strong activation. The data was obtained from mouse #0300 86 days after the cranial window surgery.

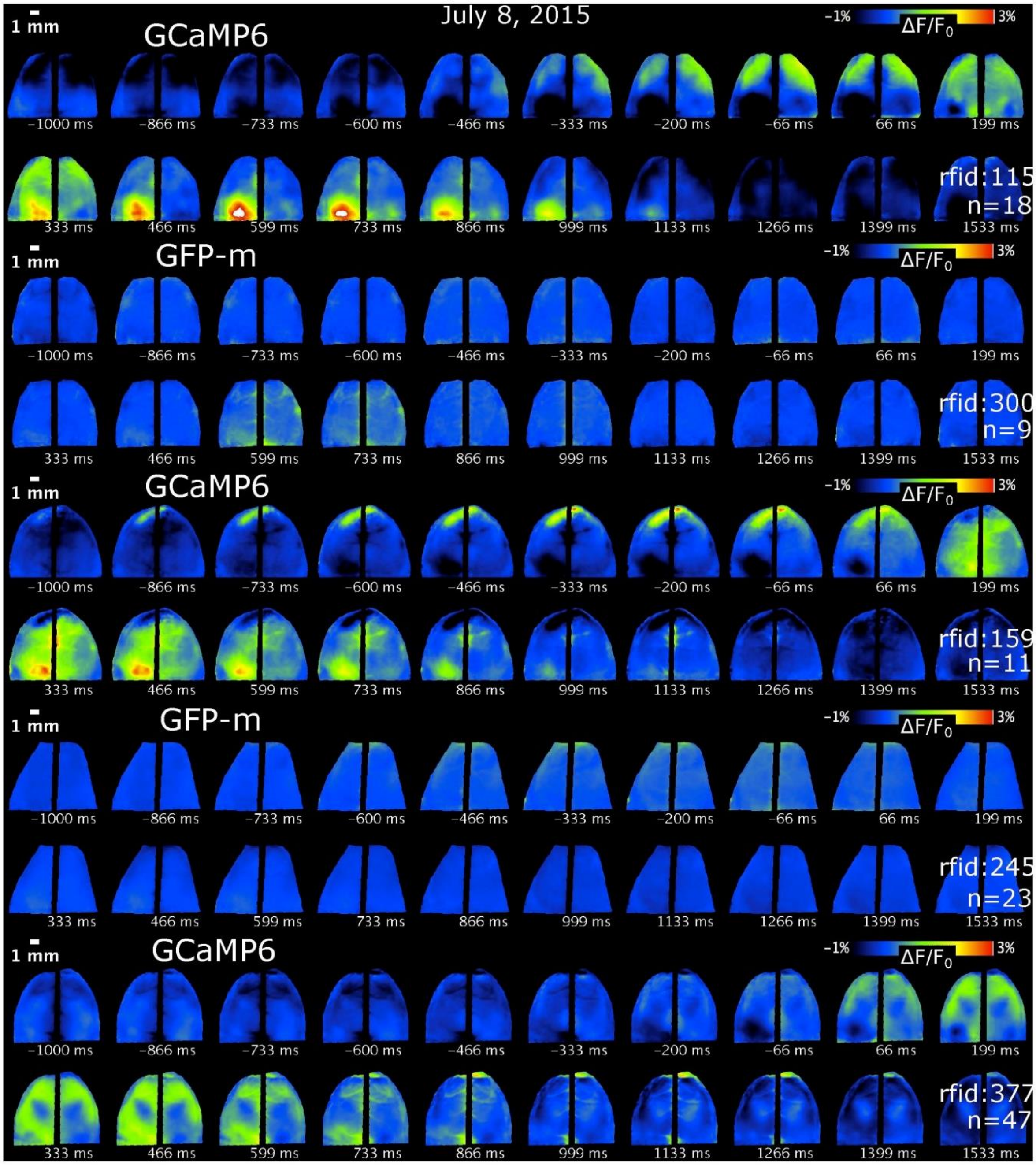

Supplementary Figure 7. Visual stimuli brain response montages. Averaged visual responses across several trials for three GCaMP6s (#0115, 0159, and 0377) and two GFP (#0245 and 0300) mice. The mice were stimulated with a 10ms 390nm LED flashes on their right eye 5 times per trial and then all the responses were averaged together for each mouse. The 390 nm LED was placed in the lateral part of the visual field that may lead to a more medial response when compared to LED placement more anterior of the mouse (Fig. 5 and Supplementary Figure 8). A strong response was observed in the left visual cortex of all the GCaMP6 mice. Little to no response is observed for the GFP-m mice. The data was obtained 60 days after the cranial window surgery.

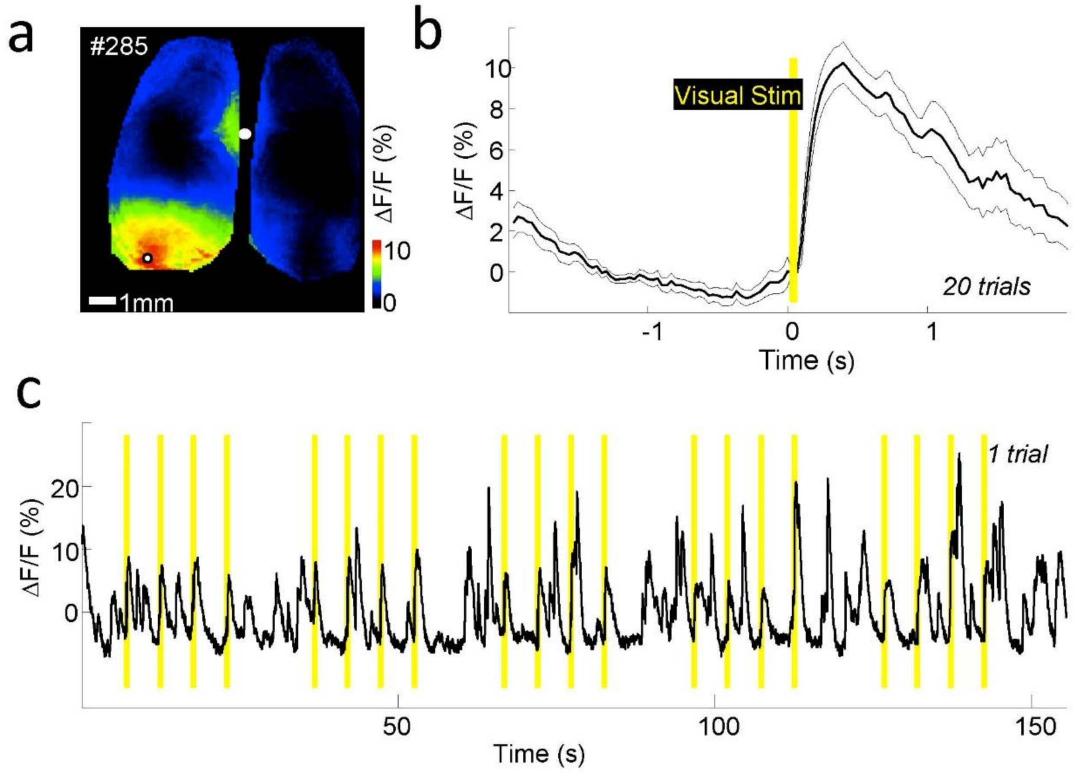

Supplementary Figure 8 Averaged visual response during prolonged episodes of head-fixation. Mouse #0285 GCaMPs single trial with 20 yellow 10 ms flash stimuli directed to the right eye acquired during a single continuous long trial, p=0.00010218 flash response versus baseline variation, n=20 responses (error indicates SEM across trials). The LED was placed anterior of the mouse at 0 degrees of elevation, white dot marks bregma. The yellow bars indicate the time of the flash stimuli.
